# Robust and Long-Lasting Immunity and Protection in Mice Induced by Lipopolyplex-Delivered mRNA Vaccines Expressing the Prefusion Protein of Respiratory Syncytial Virus

**DOI:** 10.3390/vaccines13010093

**Published:** 2025-01-20

**Authors:** Xuchang Shan, Ruiwen Han, Xueting Cheng, Jialuo Bing, Zhenyong Qi, Shucai Sun, Tangqi Wang, Qiaohong Chu, Yao Deng, Desheng Zhai, Wenjie Tan

**Affiliations:** 1 School of Public Health, Xinxiang Medical University, Xinxiang 453003, China; 13675525532@163.com (X.S.); 18755864920@163.com (J.B.); 13626457023@163.com (Z.Q.); 2 Zhejiang Provincial Key Laboratory of Medical Genetics, School of Laboratory Medicine and Life Sciences, Wenzhou Medical University, Wenzhou 325035, China; 18267839995@163.com (R.H.); wangtangqi00@163.com (T.W.); 3 National Key Laboratory of Intelligent Tracking and Forecasting for Infectious Diseases, National Institute for Viral Disease Control and Prevention, China CDC, 155 Changbai Road, Beijing 102206, China; chengxuetingabc@163.com (X.C.); chuqiaohong1999@163.com (Q.C.); dengyao@ivdc.chinacdc.cn (Y.D.); 4 Department of Nuclear Medicine, The Second Hospital of Hebei Medical University, Shijiazhuang 050004, China; 13933189361@163.com

**Keywords:** respiratory syncytial virus, prefusion protein, mRNA vaccine, lipopolyplex platform, immune protection

## Abstract

Respiratory syncytial virus (RSV) is the leading cause of lower respiratory tract infections in infants and children. mRNA vaccines based on the lipopolyplex (LPP) platform have been previously reported, but they remain unapplied in RSV vaccine development. In this study, we developed a novel LPP-delivered mRNA vaccine that expresses the respiratory syncytial virus prefusion protein (RSV pre-F) to evaluate its immunogenicity and protective effect in a mouse model. We synthesized mRNAs with gene modification for RSV pre-F and prepared mRNA vaccines using the LPP delivery platform, referred to as RSV pre-F LPP-mRNA. RSV pre-F protein expression in mRNA vaccines was characterized in vitro. Then, we evaluated the effects of the immune response and protection of this mRNA vaccine in mice up to 24 weeks post-vaccination. Following booster immunization, robust and long-lasting RSV pre-F-specific IgG antibodies were detected in the serum of mice, which exhibited Th1/Th2 balanced IgG response and cross-neutralizing antibodies against different subtypes (RSV A2, B18537, and clinical isolate hRSV/C-Tan/BJ 202301), with a clear dose–response relationship observed. RSV pre-F-specific IgG antibodies were maintained in the mice for an extended period, lasting up to 18 weeks post-immunization. Concurrently, multifunctional RSV F-specific CD8^+^ T cells (IFN–γ, IL-2, and TNF-α) were detected in the mice. After RSV A2 challenge, the RSV pre-F LPP-mRNA vaccine led to a significant reduction in viral replication, while reduced pathological damage was observed in lung tissue. The LPP-delivered mRNA vaccine expressing RSV pre-F induces a robust and long-lasting immune response and protection, indicating good prospects for further development and application.

## 1. Introduction

Respiratory syncytial virus (RSV) is a prominent global pathogen and the primary cause of acute lower respiratory tract infections in infants and young children. An estimated 33 million cases of RSV-associated acute lower respiratory tract infections were reported in children aged 0–60 months in 2019, imposing a substantial healthcare burden [1]. In addition, the disease burden of RSV in older adults is underestimated. Older adults face an increased risk of RSV-related complications and mortality owing to immunosenescence and underlying diseases [2]. In 2023, two pre-fusion (pre-F) subunit vaccines from GSK and Pfizer were approved by the US Food and Drug Administration (FDA) for use in adults or older people [3,4]. Currently, no RSV vaccines are approved for use in China.

RSV is a negative-sense, single-stranded RNA virus classified within the family Pneumoviridae and the genus Orthopneumovirus. The RSV genome is approximately 15 kb and encodes 11 distinct proteins [2]. The RSV fusion (F) protein is the main antigen responsible for inducing neutralizing antibodies [5,6].This protein displays a notable degree of conservation across various strains, rendering it a promising target for vaccine development. The F protein adopts two distinct conformations: pre-fusion (pre-F) and post-fusion (post-F). Previous reports have shown that a highly neutralization-sensitive epitope, antigenic site Ø, is found only on pre-F. And the amount of site Ø-specific antibodies was found to be correlated with NT activity. Furthermore, neutralizing antibodies found in human serum are predominantly induced by pre-F [7,8]. Therefore, pre-F is an important target for developing RSV antiviral drugs and vaccines.

As a rapidly developing vaccine platform, mRNA vaccines can be quickly developed and mass-produced in cell-free environments, offering significant prospects for widespread application [9]. These vaccines play an important role in controlling the spread of COVID-19 and have facilitated the development of vaccines targeting various respiratory pathogens. Owing to the inherently unstable nature of RNA, a safe and efficient delivery system is required to protect mRNA from degradation [10]. The currently marketed mRNA-1345, which encodes the membrane-anchored RSV pre-F protein, achieved the primary efficacy endpoints in Phase 3 trials in older adults using the same lipid nanoparticles (LNPs) as the Moderna COVID-19 vaccine [6]. However, after intramuscular injection, LNPs are not completely restricted to muscle tissue but are rapidly distributed throughout the body, accumulating in large quantities in the liver. Systemic exposure to mRNA-LNPs may lead to off-target transgene expression, leading to adverse effects such as inflammation, allergy reactions, and hemolysis. Additionally, overexpression in the liver may lead to potential adverse reactions [11]. However, to avoid these problems, we used a lipopolyplex (LPP) platform with a core–shell structure to prepare mRNA vaccines. The LPP delivery platform contains polymer-coated mRNA molecules encapsulated within a bilayer phospholipid shell. LPP expresses mRNA predominantly at the site of intramuscular injection and preferentially in the spleen rather than in the liver after vascular leakage. Targeted expression may mitigate concerns regarding potential off-target effects and systemic toxicity. Lipid–polymer hybrid particles (LPPs), a pivotal component of vaccine formulations, demonstrate excellent colloidal stability, high encapsulation efficiency, and optimal biodistribution pattern. LPP mRNA enhances the activation of the TLR7/8 signaling pathway in dendritic cells, leading to the secretion of type I interferons [12]. IFN levels in nasal washes and peripheral blood mononuclear cells (PBMCs) in adults and children negatively correlate with disease severity [13]. Thus, LPP-mRNA vaccines may induce robust antiviral responses. COVID-19 mRNA vaccines based on the LPP platform have been previously reported [12], but they remain unapplied in RSV vaccine development.

In this study, we developed an LPP-based mRNA vaccine encoding the RSV pre-F protein with genetic modifications and subsequently assessed its humoral and cellular immune responses in murine models. Furthermore, the long-lasting protective effects induced by two doses of the vaccine were compared in mice at 24 weeks post-vaccination.

## 2. Materials and Methods

### 2.1. Cells, Viruses, and Animals

The Hep-2 and HEK-293T cells used in this study were all cultured in Dulbecco’s minimal essential medium (Gibco™, Waltham, MA, USA) supplemented with 10% fetal bovine serum (PAN, Cat:P30-3302) and 1% penicillin–streptomycin (PS, Cat:15140-122). The RSV A2 strain, B18537 strain, and the clinical strain hRSV/C-Tan/BJ 202301 were isolated from Beijing in 2023 and preserved at our lab. Female Balb/c mice (6–8 weeks old, SPF grade) were purchased from Beijing Sibeifu Biotechnology Co., Ltd. (Beijing, China), and they were reared at the Animal Center of Beijing Kexing Vaccine Co., Ltd. (Beijing, China). The RSV A2 strain, B18537 strain, and the clinical strain hRSVC-TanBJ 202301 were maintained at the Institute for Viral Disease Control and Prevention, Chinese Center for Disease Control and Prevention. Female Balb/c mice (6–8 weeks old, specific pathogen-free grade) were procured from Beijing Sibeifu Biotechnology Co., Ltd. (Beijing, China) and housed at the Animal Facility of Beijing Kexing Vaccine Co., Ltd. (Beijing, China). All animal experiments were conducted according to ethical regulations and were approved by the local ethics committee (Animal Ethics No. 20231128092).

### 2.2. Construction and Preparation of mRNA Vaccine Expressing RSV Pre-F Protein

**Figure 1:**
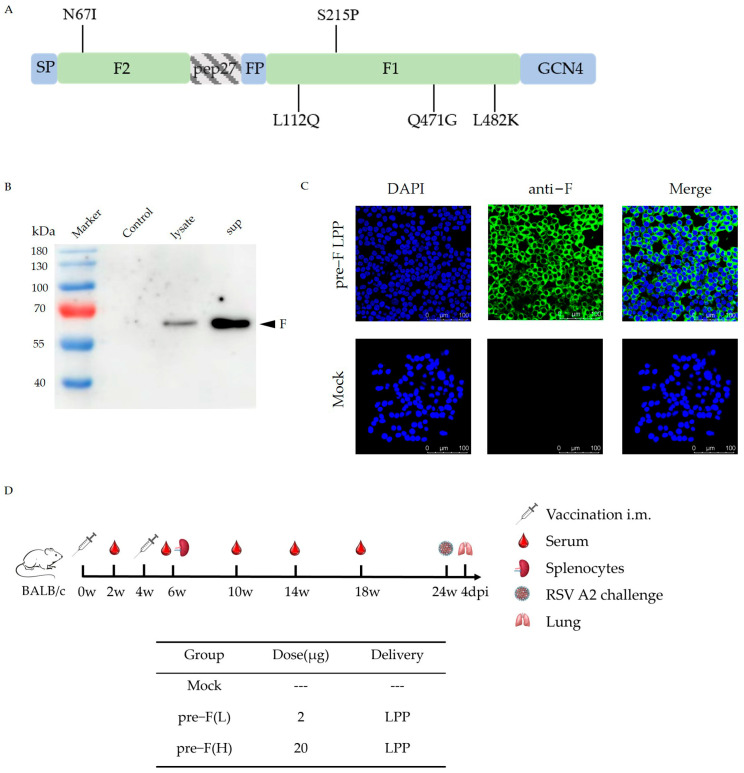
Characterization of the RSV pre-F LPP-mRNA vaccines and scheme of the animal experiment in this study: (**A**) construction of RSV pre-F; (**B**) pre-F protein expression in mRNA vaccines was detected via Western blot in HEK-293T cells transfected with the RSV pre-F mRNA; (**C**) pre-F protein expression in mRNA vaccines was detected via immunofluorescence in HEK-293T cells transfected with the RSV pre-F mRNA; (**D**) immunization and challenge schema in mice for the RSV pre-F LPP-mRNA vaccines.

The recombinant RSV pre-F protein was designed with reference to the relevant literature [14,15]. Peptide 27 was deleted, while a Lys residue at one of the two furin sites was conserved. Mutations were introduced at L112Q, Q471G, and L482K to stabilize the F protein in the pre-F conformation and reduce protein hydrolysis [14,15]. Additionally, mutations at N67I and S215P increased protein expression levels [14,15]. The transmembrane structural domain and the cytoplasmic region at the C-terminal of the F protein were substituted with the GCN4 trimeric motif to stabilize the F protein as a trimer. The pre-F sequences were synthesized via codon optimization and RSV pre-F-mRNA with LPP encapsulation by Shanghai Stemirna, including the composition of the lipid shells and the characteristics of the particles such as size; the zeta potential of the lipid-based nanoparticles has been described previously [12,16,17,18] (Figure 1A).

### 2.3. Western Blotting

To analyze the expression of RSV pre-F protein, HEK-293T cells were transiently transfected with RSV pre-F-mRNA, and the resulting supernatant and cell lysate were collected 6 h post-transfection. The proteins were harvested using lysis buffer and then boiled for 10 min. Denatured samples were resolved with 10% sodium dodecyl sulfate-polyacrylamide gel electrophoresis (SDS-PAGE) and subsequently transferred onto nitrocellulose membranes. An anti-RSV polyclonal antibody from Abcam (Cambridge, UK) was utilized at a dilution of 1:1000, followed by a horseradish peroxidase-conjugated rabbit anti-goat IgG secondary antibody from ZSGB-BIO (Beijing, China) at a dilution of 1:5000. The membranes were visualized using a chemiluminescent substrate and analyzed with a chemiluminescent imaging system (Figure 1B).

### 2.4. Immunofluorescence Assay

HEK-293T cells were cultured in Dulbecco’s modified Eagle’s medium (Hyclone, South Logan, UT, USA) supplemented with 10% fetal bovine serum (Gibco, Grand Island, NY, USA) and 1% penicillin–streptomycin (Gibco, Grand Island, NY, USA) in a 5% CO_2_ incubator at 37 °C. The HEK-293T cells were transiently transfected with RSV pre-F-mRNA (no transfection reagents were used in this case, and LPP particles without RSV mRNA served as a relevant control). Subsequently, the cells were fixed in pre-cooled 4% paraformaldehyde, permeabilized in 0.2% Triton X-100, and blocked with 10% goat serum in phosphate-buffered saline. The primary antibody used was an anti-RSV F protein monoclonal antibody from Sino Biological (Beijing, China). Following incubation, the cells were washed and incubated with secondary antibodies (fluorescein isothiocyanate (FITC)-labeled goat anti-rabbit IgG from ZSGB-BIO, China) at room temperature for 1 h. The samples were then washed and incubated with 4′,6-diamidino-2-phenylindole (DAPI, Beyotime, Shanghai, China) for 10 min. Fluorescent images were captured using a Leica TCS SP8 confocal microscope (Leica Biosystems, Wetzlar, Germany) (Figure 1C).

### 2.5. Immunizations

Mice were divided randomly into three groups and immunized with 20 μg (H) and 2 μg (L) of RSV pre-F LPP-mRNA, respectively. The control group was injected with LPP particles without RSV mRNA. The mice were immunized twice at 4-week intervals. Blood samples were collected 2 weeks after each immunization to analyze the humoral immune response. Splenocytes were isolated 2 weeks after the booster immunization to analyze the cellular immune response. Additional blood samples were collected at 10, 14, and 18 weeks post-prime to evaluate long-lasting immunity (Figure 1D).

### 2.6. Enzyme-Linked Immunosorbent Assay (ELISA)

To detect humoral immune response, ELISA was performed as previously described [19]. Briefly, plates (Corning, Shanghai, China, Asia) were coated with RSV F protein (Sino Biological, China) overnight at 4 °C. After blocking with 10% goat serum in PBS for 2 h at 37 °C, a serially diluted mouse serum was added, and the plates were then incubated for 1 h at 37 °C. Subsequently, a goat anti-mouse IgG horseradish peroxidase (HRP)-conjugated secondary antibody was then applied, and the plates were incubated for an additional 1 h at 37 °C. The immune reaction was developed using a tetramethylbenzidine (TMB) substrate-based assay. The reaction was stopped with 2M H_2_SO_4_ and measured at 450 nm using a plate reader (Multiskan MK3, Thermo Fisher Scientific, Waltham, MA, USA). Values that exhibited a 2.1-fold increase compared to the control group were considered positive. For IgG antibody subtyping, the antigen was coated overnight at 4 °C as described above. After blocking, mouse serum was added in serial dilutions and incubated for 1 h at 37 °C. Secondary antibodies, biotinylated anti-mouse IgG1 (mAb MG1), and IgG2a (mAb MG2a) (MABTECH, Nacka, Sweden), were applied for 1 h. Streptavidin-HRP was then added, and the mixture was incubated at 37 °C for 1 h. Color development, termination, and microplate reader measurement were performed as described above.

### 2.7. RSV Neutralisation Assays

Serial dilutions of heat-inactivated sera were mixed with cell culture medium containing RSV—strain A2, B18537, or clinical isolate hRSV/C-Tan/BJ 202301—and incubated for 1 h at 37 °C. The mixture was subsequently inoculated onto a HEp-2 cell monolayer in a 96-well plate and incubated for 1 h at 37 °C. Following removal of the mixture, a methylcellulose overlay was applied, and the plate was further incubated at 37 °C for 48 h. The plates were then treated with anti-RSV G protein monoclonal antibody (Vazyme, Nanjing, China). Fluorescently labeled viral foci were enumerated using a CTL Immunospot Analyser (Cellular Technology Ltd., Cleveland, OH, USA). NT50 was calculated as the reciprocal serum dilution at which 50% of the virus was neutralized compared to those of the control wells without serum.

### 2.8. Enzyme-Linked Immunospot Assay (ELISPOT)

Two weeks after the booster immunization, mice from each group were randomly selected and sacrificed. Spleens were harvested for IFN-γ ELISPOT analysis, as previously described [20]. Briefly, 96-well enzyme-linked immunospot (ELISPOT) plates were coated overnight at 4 °C with a purified anti-mouse interferon-gamma (IFN-γ) capture antibody (BD ELISPOT Set, BD Biosciences, Franklin Lakes, NJ, USA). Splenocyte suspensions from each experimental group were stimulated with RSV F peptides (KYKSAVTEL, TYMLTNSEL, and GWYTSVITIELSNIK) [21,22] or Concanavalin A (ConA) as a positive control. Subsequent procedures were carried out as per the manufacturer’s instructions. Spot-forming units (SFUs) were used to quantify IFN-γ-secreting T cells using an ELISpot plate reader from Biosys (South Pasadena, CA, USA).

### 2.9. Intracellular Cytokine Staining (ICS)

RSV F peptides and splenocyte suspensions were added to the flow tube, and cytokine production by memory T cells was evaluated through surface and intracellular cytokine staining using a Fixation/Permeabilization Solution Kit (BD Biosciences, Franklin Lakes, NJ, USA). Fluorochrome-conjugated antibodies used for surface staining included PerCP-Cy™5.5 Hamster Anti-Mouse CD3e, BV510 Rat Anti-Mouse CD4, FITC Rat Anti-Mouse CD8a, BV421 Rat Anti-Mouse IFN-γ, PE-Cy™7 Rat Anti-Mouse TNF, PE Rat Anti-Mouse IL-2, and APC Rat Anti-Mouse IL-4. The frequency of F protein-specific T cells was determined using FACS II and analyzed using FlowJo software (version 10.8.1).

### 2.10. Challenge Experiment

At 20 weeks after the booster immunization, the mice were challenged with 2 × 10^6^ PFU of RSV A2 in 50 μL via intranasal infection. Lung tissues were collected at 4 dpi (Figure 1D). Half of the tissues were used to determine viral load and RNA copy number as previously described [23]. The remaining half of the sample was fixed in a 4% formalin solution and processed into hematoxylin and eosin (H&E)-stained sections for subsequent histopathological evaluation.

### 2.11. Statistical Analysis

The unpaired non-parametric Mann–Whitney test was employed for comparisons between two groups, while one-way analysis of variance (ANOVA) was utilized for comparisons among multiple groups. Data are presented as the mean ± standard error of the mean (SEM). Statistical analyses were performed using GraphPad Prism 9.0 software (GraphPad Software, Boston, MA, USA). Asterisks in the figures indicate the level of statistical significance (* *p* < 0.05, ** *p* < 0.01, and *** *p* < 0.001).

## 3. Results

### 3.1. Characterisation of RSV Pre-F LPP-mRNA Vaccines

Figure 1A depicts the structure of the RSV pre-F protein. HEK-293T cells were transfected with RSV pre-F mRNA, and the expression of the pre-F protein was confirmed through Western blot analysis, as shown in Figure 1B. The pre-F protein exhibited a molecular weight of approximately 56.7 kDa. Target bands were observed at the corresponding positions. Moreover, pre-F protein expression in mRNA vaccines was detected via immunofluorescence in HEK-293T cells transfected with the RSV pre-F mRNA (Figure 1C).

### 3.2. Significant and Sustained Humoral Immunity Responses Induced by RSV Pre-F LPP-mRNA

**Figure 2:**
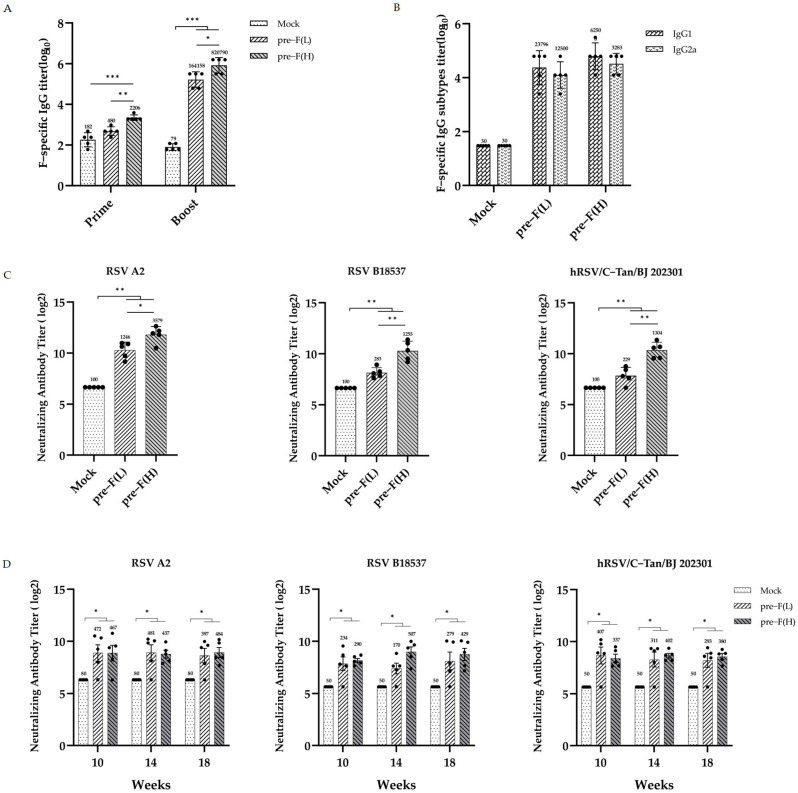
RSV pre-F LPP-mRNA vaccines induced long-lasting F-specific IgG antibody responses and high neutralization antibody responses in mice. (**A**) ELISA analysis of RSV F-specific IgG antibodies (*n* = 5). (**B**) ELISA analysis of the IgG subtype (*n* = 5). (**C**) Neutralizing antibody titers against RSV A2, B18537, and clinical isolate hRSV/C-Tan/BJ 202301, measured after booster immunization (*n* = 5). (**D**) Long-term neutralizing antibody titers against RSV A2, B18537, and clinical isolate hRSV/C-Tan/BJ 202301 (*n* = 5). The statistical comparisons between groups were performed using two-way analysis of variance (ANOVA) and unpaired non-parametric Mann–Whitney tests. The bars plotted show the means ± SEM. The results represent three independent experiments (* *p* < 0.05, ** *p* < 0.01, and *** *p* < 0.001).

Immunization and challenge schema in mice for the RSV pre-F LPP-mRNA vaccines are illustrated in Figure 1D. The serum of the mice was collected at 2, 6, 10, 14, and 18 weeks post-initial immunization for humoral immune response detection. The results showed that certain levels of F-specific IgG antibodies were detected in the vaccinated group as early as 2 weeks after initial immunization. The IgG levels in the high-dose group were significantly higher than those in the control group. Booster immunization significantly increased the IgG levels in all immune groups, with the levels significantly higher than those in the control group (Figure 2A, left). Additionally, we measured the levels of specific IgG1 and IgG2a antibodies in the serum of mice 2 weeks after booster immunization. The IgG1 antibody titer induced by all immune groups was slightly lower than that of IgG2a; however, no significant difference was observed between the groups (Figure 2B). These findings suggest that pre-F LPP-mRNA vaccines induce a more balanced Th1/Th2 immune response.

To evaluate the neutralization activity in mice. Following booster immunization, sera from mice were detected using neutralization assays using different RSV strains. The data show that both the high- and low-dose immunization groups produced high levels of neutralizing antibodies against two subgroups of RSV strains (A2 and B18537 strains) (*p* < 0.01). Furthermore, high levels of neutralizing antibodies were also produced against the clinical isolates hRSV/C-Tan/BJ 202301, which belong to subgroup ON1 and are currently prevalent in China, indicating that the pre-F LPP-mRNA vaccine provides extensive protection against different RSV strains (Figure 2C).

To further evaluate the long-lasting humoral response, serum neutralization activity against different RSV strains was detected in mice at weeks 10, 14, and 18 after vaccination. Neutralizing antibody titers in each immunized group decreased but remained significantly higher than those in the control group (*p* < 0.001) from weeks 10 to 18, with no significant difference observed between the two different doses of immunization groups (Figure 2D). These findings indicate that the 2 μg dose of pre-F LPP-mRNA is sufficient to elicit a robust and long-lasting humoral immune response.

Moreover, we investigated the dose dependence of the vaccine. At 2 weeks following the prime and booster immunizations, the higher dose of mRNA induced significantly higher levels of IgG antibodies than the lower dose, with a dose-dependent effect (Figure 2A). The pre-F (H) also demonstrated significantly higher levels of neutralizing antibodies against various strains 2 weeks after the booster immunizations (Figure 2C). However, strong neutralizing antibody levels were maintained in mice with pre-F LPP-mRNA at weeks 10–18 after vaccination. Collectively, these findings suggest that pre-F LPP-mRNA induces a potent humoral immune response in mice.

### 3.3. RSV Pre-F LPP-mRNA Elicits Significant F-Specific T Cell Responses

**Figure 3:**
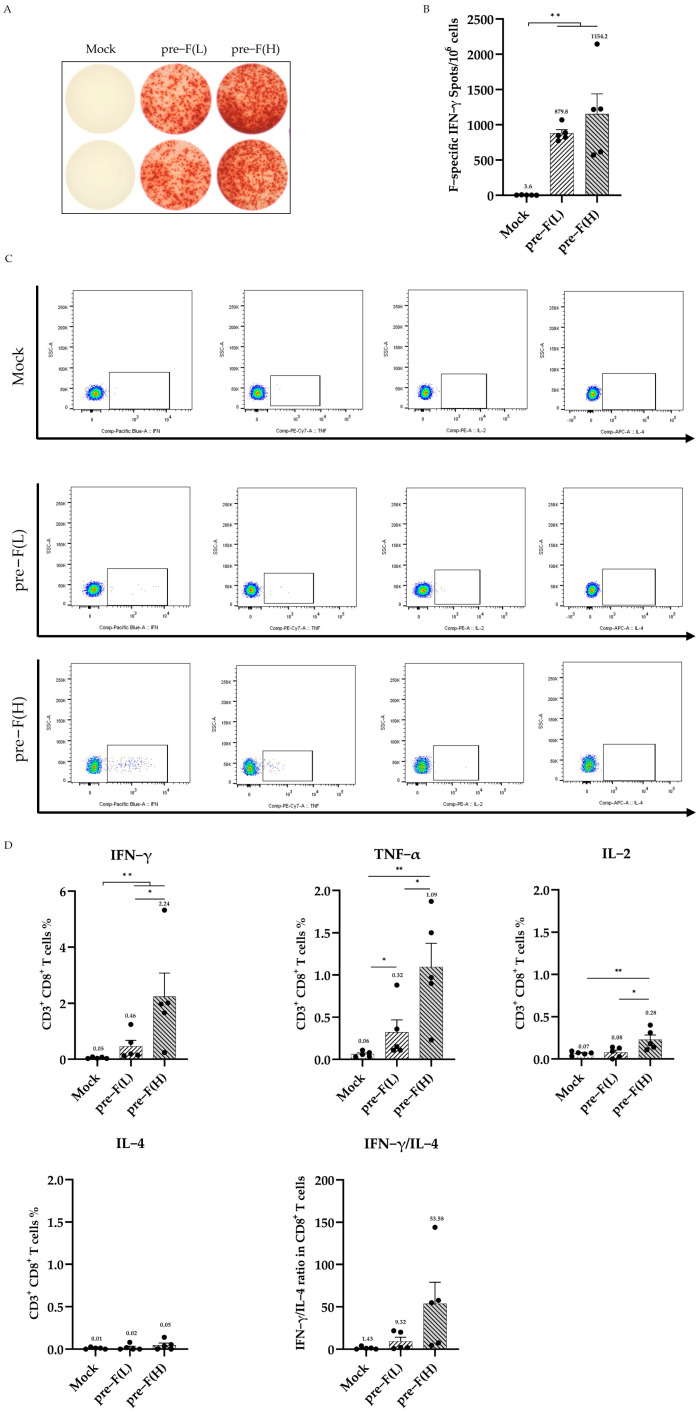
Th1-biased T-cell responses induced by RSV pre-F LPP-mRNA vaccines: (**A**) representative images from ELISpot assays; (**B**) quantification of splenocytes secreting IFN-γ using ELISpot assays (*n* = 5); (**C**) representative flow cytometry plots illustrating the expression of intracellular cytokines by CD8^+^ T cells; (**D**) percentages of RSV F-specific cytokine-positive cells within the total CD8^+^ T cell populations (*n* = 5). Statistical comparison among groups was performed using two-way ANOVA followed by Tukey’s multiple comparison test (* *p* < 0.05 and ** *p* < 0.01).

To evaluate the T cell responses induced by the pre-F LPP-mRNA vaccine in mice, splenocytes from vaccinated mice were stimulated with F-specific peptides, and an ELISpot assay was performed to quantify the number of F-specific T cells. Following booster immunization, the high- and low-dose mRNA vaccine groups showed significantly higher levels of IFN-γ secretion than the control. While the number of IFN-γ-secreting T cells in the RSV pre-F (L) was lower than that in the RSV pre-F (H), this difference was not significant (Figure 3A,B). The 2 μg dose of pre-F LPP-mRNA is sufficient to induce high levels of F-specific T cell immune responses in mice.

The T cell response was further assessed using an intracellular cytokine staining assay (ICS). Following booster immunization, all groups with pre-F LPP-mRNA exhibited significantly elevated levels of CD8^+^ T cells primarily secreting Th1-type cytokines, including IFN-γ, IL-2, and TNF-α, with no detectable secretion of the Th2. Additionally, high-dose immunity induced stronger cytokine secretion in CD8^+^ T cells, showing a dose-dependent effect (Figure 3C,D). However, no significant level of cytokine secretion was detected using ICS in CD4^+^ T cells from immunized mice (Supplemental Figure S1). These findings suggest that pre-F LPP-mRNA vaccination induces a specific and effective CD8^+^ T cell immune response in mice.

### 3.4. RSV Pre-F LPP-mRNA Induced Long-Lasting Protection Against RSV Infection in Mice

**Figure 4:**
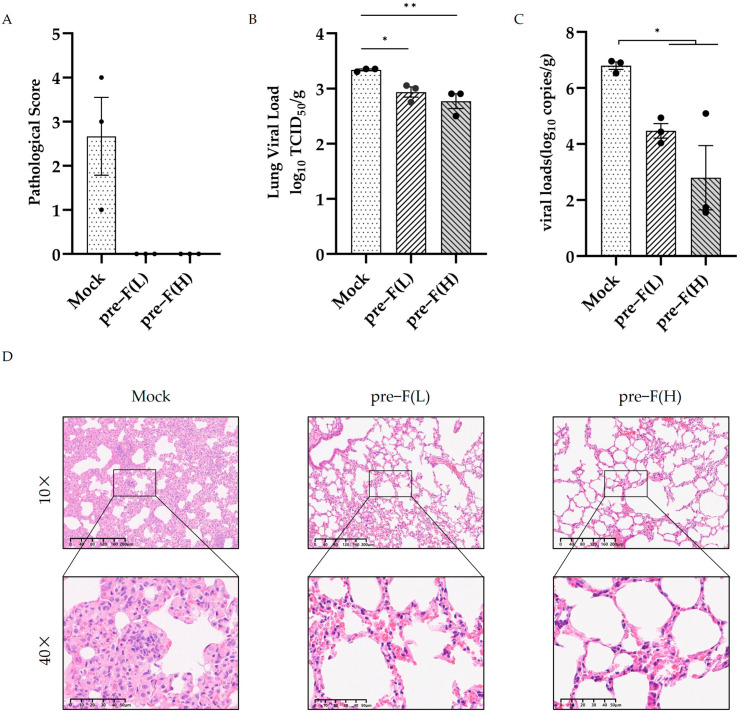
The RSV pre-F LPP-mRNA vaccine protects RSV A2 in mice. (**A**) Lung injury pathological scores (*n* = 3). (**B**) Viral TCID50 in lung tissues post-challenge (*n* = 3). (**C**) Viral RNA load in lung tissues of mice following infection (*n* = 3) detected using real-time RT-PCR. (**D**) Hematoxylin and eosin (HE) staining results of lung tissue sections post-challenge. Statistical significance was determined using the two-tailed Mann–Whitney test (* *p* < 0.05, and ** *p* < 0.01).

Given that vaccine immunization induces a strong and long-lasting immune response in mice, we further explored the protective effect of the pre-F LPP-mRNA vaccine against RSV infection. At 24 weeks post-vaccination, the mice were challenged with (RSV A2, 2 × 10^6^ PFU/piece), and their lungs were collected for analysis 4 days after RSV exposure. The pathological score of lung injury showed that the pre-F LPP-mRNA-immunized group had significantly better lung pathology than those in the mock group (Figure 4A). Additionally, mice in the control groups showed significantly higher viral loads in the lungs than those in pre-F LPP mRNA-immunized groups (Figure 4B). Viral copy numbers using real-time RT-PCR showed similar results, with higher levels of viral copies detected in the mock group (Figure 4C).

Histopathological examination of the lung tissue from the mice showed degeneration, necrosis, and detachment of alveolar epithelial cells, along with nuclear fragmentation (Figure 4D). Additionally, many lymphocytes, macrophages, neutrophils, and other inflammatory cells infiltrated alveolar cavities and widened the interstitial space. The control group showed significant proliferation of type II alveolar epithelial cells. In contrast, the pre-F LPP-mRNA-immunized mice exhibited normal alveolar epithelial morphology, with intact connections between adjacent flat epithelial cells. The bronchiole intima was intact, with no exudates observed in the alveoli or bronchiole lumen.

## 4. Discussion

Recently, an RSV mRNA vaccine based on an LNP-delivered platform was also approved by the FDA for protecting adults aged ≥60 years [6]. However, more safe and potent mRNA platforms and candidates still need to be explored. In this study, a novel LPP-mRNA vaccine expressing RSV pre-F protein (RSV pre-F LPP-mRNA) was constructed. This vaccine induced specific humoral immune responses and T cell immune responses against RSV F protein in mice, as well as significantly enhanced cytokine secretion in CD8^+^ T cells. The humoral immunity generated by this vaccine showed broad-spectrum neutralizing activity and high persistence, while mice remained protected against RSV A2 infection 24 weeks after initial immunization.

High levels of neutralizing antibodies are associated with protection against severe RSV disease. Moreover, their levels are inversely correlated with the risk of RSV reinfection [24,25,26]. Therefore, an ideal RSV vaccine should induce high levels of neutralizing antibodies. The pre-F as the vaccine antigen can produce relatively higher levels of neutralizing antibodies, which are expected to provide potent protection. In this study, high levels of protective neutralizing antibodies against RSV laboratory-adapted strains (A2 and B18537) were induced in all immunized mice after the boost. However, since these laboratory strains were isolated before 1970 and cultured in successive laboratory passages, they may not accurately reflect the immune response and genetic composition of the current RSV epidemic strains [27]. Therefore, we examined the neutralizing effect of mouse serum post-booster immunization against a clinical isolate of hRSV/C-Tan/BJ 202301. The results showed that the high- and low-dose vaccine immunization groups produced neutralizing antibodies against the clinical isolates, and the high-dose immunization group produced more significant neutralizing activity.

However, neutralizing antibodies alone may not provide sufficient protection against RSV disease or reinfection in some individuals, nor do they offer universal durable immune protection [28]. CD8^+^ T cells play a crucial role in viral clearance by secreting cytokines, directly interacting with infected cells, and modulating immune responses to prevent excessive tissue damage [29,30,31]. Depletion of CD8^+^ T cells in RSV-infected mice in murine models led to a delay in viral clearance compared to that observed in control mice, whereas the transfer of CD8^+^ T cells from previously RSV-infected mice to naive mice resulted in decreased weight loss and viral load following RSV infection [30].The results of this study show that the high-dose and low-dose vaccine groups induced the production of IFN-γ-secreting T cells, thereby significantly enhancing cellular immunity. Additionally, ICS was used to assess cytokine secretion by specific CD8^+^ T cells in mouse lymphocytes following peptide stimulation. The results showed that CD8^+^ T cells mainly secreted IFN-γ, IL-2, and TNF-α, indicating that the vaccine induces a Th1-biased immune response. This Th1-skewed response reduces the possibility of a Th2-biased immune reaction, leading to more severe disease and immunopathology [32].

RSV outbreaks exhibit a seasonal pattern globally, typically occurring from November to April in the Northern Hemisphere and from March to October in the Southern Hemisphere [33]. In contrast, the antibody response generated by natural RSV infection is short-lived. Healthy adults can be repeatedly infected with the same RSV strain for 2 months, with a 25% risk of reinfection even among those with the highest antibody levels [28]. Therefore, a vaccine that induces long-lasting immunity may provide effective protection throughout the RSV epidemic season. However, studies on the long-lasting protective effects of RSV vaccines remain limited. Therefore, we investigated the long-lasting immune effect of high- and low-dose LPP-mRNA vaccines. The results showed that the humoral immunity induced by the LPP-mRNA vaccine remained stable in vivo for a long time, exhibiting strong neutralizing activity against RSV strains A and B and clinical isolates. Following the challenge, the immune group continued to effectively inhibit RSV replication in the lungs and reduce pathological injury to the lungs. Furthermore, the low-dose group also exhibited effective protection against RSV challenge at least 24 weeks after vaccination. This protection may be attributed to the high levels of neutralizing antibodies induced in the low-dose group over the long-term immunization process compared to those observed in the high-dose group.

This study had some limitations, including the fact that only the RSV A strain was tested, and the protective effect of the vaccine can be more comprehensively evaluated by challenging it with more RSV A and B strains in subsequent experiments; in addition, the vaccine effect was evaluated only in the mouse model, and further studies can be conducted with more sensitive animal models (cotton rats, African green monkeys, etc.). Furthermore, a previous report presented evidence that the LPP-delivered mRNA vaccine enters dendritic cells and stimulates IFN-b and IL-12 expression [16]; it needs to be elucidated if robust and long-lasting immunity and protection in mice might be correlated with increased IFN-b and IL-12 in the plasma of mice induced by the LPP-RSV vaccine in this study.

## 5. Conclusions

In conclusion, the prepared LPP-mRNA vaccine expressing RSV pre-F protein induced robust and long-lasting immunity and protection in mice. Even at low doses, the vaccine effectively protected mice against the RSV A2 challenge, significantly inhibiting viral replication and reducing lung tissue pathological damage. The findings provide a scientific foundation for further development and application of this novel RSV mRNA vaccine.

## Data Availability

The datasets generated during the current study are available from the corresponding author upon reasonable request.

## Supplementary Materials

The following supporting information can be downloaded at: https://www.mdpi.com/article/10.3390/vaccines13010093/s1. Figure S1: Detection of intracellular cytokines IFN-γ, IL-2, TNF, and IL-4 in CD4+T cells from pre-F LPP-mRNA-immunized mice.
