# Supplementary material for: Robust and Long-Lasting Immunity and Protection in Mice Induced by Lipopolyplex-Delivered mRNA Vaccines Expressing the Prefusion Protein of Respiratory Syncytial Virus
Source: Vaccines (Basel). 2025 Jan 20;13(1):93. doi: 10.3390/vaccines13010093 (PMC11769257; doi:10.3390/vaccines13010093)
Supplement: Supplementary file 1 [file vaccines-13-00093-s001.zip › vaccines-3418933-supplementary.pdf]

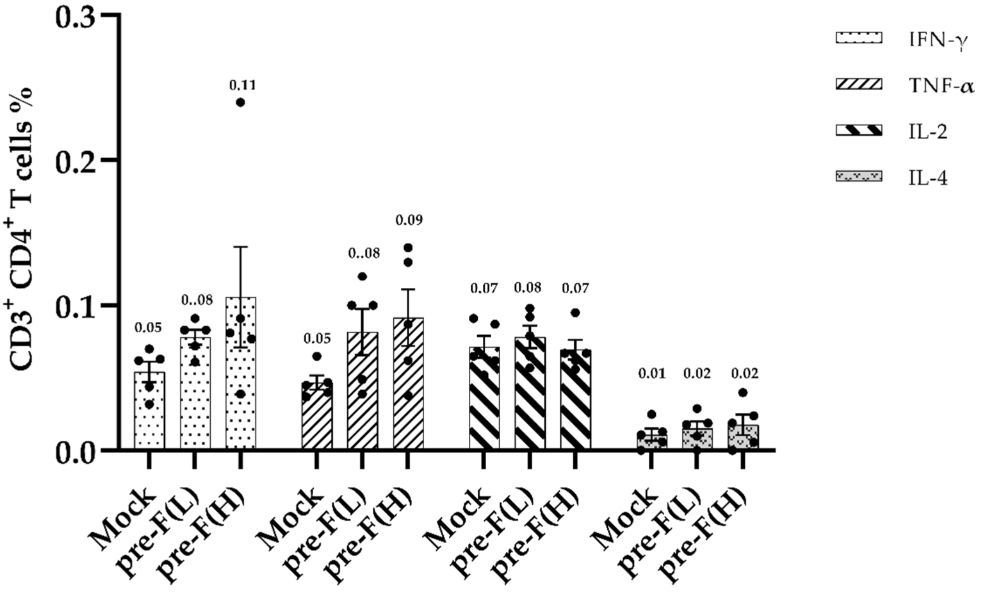

Supplementary Figure S1. Detection of intracellular cytokines IFN-γ, IL-2, TNF, and IL-4 in CD4+T cells from pre-F LPP-mRNA-immunized mice.
